# dRYBP Contributes to the Negative Regulation of the *Drosophila* Imd Pathway

**DOI:** 10.1371/journal.pone.0062052

**Published:** 2013-04-15

**Authors:** Ricardo Aparicio, Claudine Neyen, Bruno Lemaitre, Ana Busturia

**Affiliations:** 1 Centro de Biología Molecular Severo Ochoa, Consejo Superior de Investigaciones Científicas and Universidad Autónoma de Madrid, Madrid, Spain; 2 Global Health Institute, School of Life Sciences, École Polytechnique Fédérale de Lausanne, Lausanne, Switzerland; Ecole Normale Supérieur de Lyon, France

## Abstract

The *Drosophila* humoral innate immune response fights infection by producing antimicrobial peptides (AMPs) through the microbe-specific activation of the Toll or the Imd signaling pathway. Upon systemic infection, the production of AMPs is both positively and negatively regulated to reach a balanced immune response required for survival. Here, we report the function of the dRYBP (drosophila Ring and YY1 Binding Protein) protein, which contains a ubiquitin-binding domain, in the Imd pathway. We have found that *dRYBP* contributes to the negative regulation of AMP production: upon systemic infection with Gram-negative bacteria, *Diptericin* expression is up-regulated in the absence of *dRYBP* and down-regulated in the presence of high levels of dRYBP. Epistatic analyses using gain and loss of function alleles of *imd*, *Relish,* or *skpA* and *dRYBP* suggest that dRYBP functions upstream or together with SKPA, a member of the SCF-E3-ubiquitin ligase complex, to repress the Imd signaling cascade. We propose that the role of dRYBP in the regulation of the Imd signaling pathway is to function as a ubiquitin adaptor protein together with SKPA to promote SCF-dependent proteasomal degradation of Relish. Beyond the identification of dRYBP as a novel component of Imd pathway regulation, our results also suggest that the evolutionarily conserved RYBP protein may be involved in the human innate immune response.

## Introduction

Biological pathways involved in stress responses, like those associated with innate immunity, must quickly and efficiently modulate gene expression to ensure survival of the organism. *Drosophila* uses the evolutionarily conserved host defense of innate immunity to protect against microbial infection and relies mainly on the Toll and Imd pathways to regulate the expression of different AMP genes (for recent reviews see [1]–[3]). AMPs are constitutively expressed in immuno-competent epithelial tissues to defend the body against infection [4], [5]. Furthermore, upon systemic microbial infection the Toll and Imd pathways up-regulate AMP production by the fat body and blood cells. Once the infection is controlled, AMP expression is down-regulated to avoid deleterious immuno-pathological reactions [6].

The Imd signaling pathway is activated by infection with Gram-negative bacteria and Gram-positive bacilli [7]. The activation is initiated upon detection of peptidoglycan (PGN) by PGRP-LC, a member of the peptidoglycan recognition proteins, at the plasma membrane [7]–[9]. Transduction of this signal requires ligand-induced receptor oligomerization with subsequent assembly of a signaling complex containing IMD, DREDD, and dFADD receptor associated proteins [2], [10]–[12]. The activation of this pathway leads to the post-translational modification of the *Drosophila* NF-κB factor Relish, and its nuclear translocation [13]. Relish ultimately drives transcription of IMD-specific AMP genes such as *Diptericin* and *Attacin-B* as well as several regulatory Imd pathway components.

Regulation of NF-κB pathway activity in both invertebrates and vertebrates is achieved at multiple levels through ubiquitin-mediated post-translational modification of signaling components [14], [15]. In *Drosophila*, selective linkage of mono- or poly-ubiquitin chains triggering degradation or stabilization of Imd pathway components plays a crucial role in maintaining a balanced immune response. IMD first undergoes cleavage by the caspase DREDD, which itself is activated by poly-ubiquitylation [16], [17]. The cleaved IMD protein is then tagged with K63-linked poly-ubiquitin chains by the E3 ligase dIAP2 in complex with E2 conjugases Uev1a, dUbc13/Bendless and Effete [17], [18], and ubiquitylated IMD acts as an assembly platform for downstream adaptors TAB2/dTAK1 [19]. Subsequent editing of K63- to K48-linked ubiquitin chains through the ubiquitin hydrolase dUsp36 ends signaling by targeting IMD for proteasomal degradation [20]. As in vertebrates, ubiquitylation/deubiquitylation events also regulate stability of the *Drosophila* IKK complex (ird5/key) [19], [21]–[23]. DREDD-mediated cleavage of Relish is thought to be held in check by Caspar, a protein with multiple ubiquitin-related domains [24], and by DNR1, a RING-domain containing protein which binds to DREDD and has been proposed to target it for proteasomal degradation [25]. Finally, both intact and processed Relish has been suggested to undergo ubiquitin-mediated degradation through SKPA, a member of the E3-ubiquitin ligase SCF complex [26].

The *dRYBP* (*drosophila Ring* and *YY1 Binding Protein*) gene [27] encodes a protein that is evolutionarily conserved in vertebrates (known as RYBP/DEDAF/YAF2) and that contains in its N-terminus a ubiquitin-binding domain of the NZF (Nucleoporin Zinc Finger) type [28]. Studies both in vertebrates and *Drosophila* have described the phenotypic effects of high and low levels of dRYBP expression and also its interactions with several proteins involved in a range of biological processes, including epigenetic transcriptional regulation mediated by the Polycomb and trithorax groups of proteins [27], [29]–[34]. Human RYBP/DEDAF has been shown to interact with DED (Death Effector Domain) containing proteins [35], [36] that mediate homotypic interactions important for the assembly and activation of apoptotic and inflammatory complexes [37]. In *Drosophila*, high levels of dRYBP induces apoptosis in imaginal disc cells and this apoptosis is dependent on dFADD and DREDD [31], two DED containing proteins [37] also involved in the IMD-mediated immune response in *Drosophila* [10]–[12].

In this work we show that dRYBP contributes to the negative regulation of the Imd signaling pathway. Notably, we have found that dRYBP is required for the inhibition of the production of AMPs upon systemic infection. We propose that dRYBP functions in the immune response to promote ubiquitin-dependent degradation of IMD-pathway components, among those the Relish protein.

## Materials And Methods

### Fly stocks

*Canton^S^* were used as control flies. *dRYBP* null allele stocks were *dRYBP^1^/CyO GFP* and *dRYBP^Δ55^/ CyO GFP* [30]. Deficiencies uncovering the *dRYBP* genomic region were *Df(2R)BSC598* and *Df(2R)BSC787* (http://flybase.bio.indiana.edu). The GAL4 lines used were *c564-Gal4*, *en-Gal4*, *hs-Gal4* (http://flybase.bio.indiana.edu). The UAS lines used were: *UAS-dRYBP, UAS-dRYBP_RNAi_* [30], *UAS-imd* [38], *UAS-skpA_RNAi_* (http://www.flyrnai.org/TRiP-HOME.html), *UAS-Relish-His-6* [39], *pUASt-Venus-dRYBP* (this work). The *UAS-VDRC_RNAi_* lines [40] were obtained from http://stockcenter.vdrc.at.

### Adult infection

The Gram-negative entomopathogen *Erwinia carotovora carotovora 15* (*Ecc15*), and the Gram-negative *Escherichia coli* (*E. coli*) were grown overnight as shaking cultures in LB medium at 29°C (*Ecc15*) and 37°C (*E. coli*) and pelleted to an OD_600_ of 200 (*Ecc15*) or to an OD_600_ of 400 (*E. coli*). Systemic infection of flies was done by pricking a needle dipped in a bacterial pellet into the thorax of 2–5-day old adult females. Where appropriate, pathogens were heat-killed at 95°C during 15 min.

### Cloning and generation of transgenic flies

The coding sequence of *dRYBP* (BDGP DGC clone LD18758) without start codon was cloned into pENTR-D-TOPO (Life Technologies) using the following primers: Forward: 5′-CACCGACAAGAAATCCTCGCCG- 3′, Reverse: 5′-CTAACTCCGGCTGTCGTTG-3′. The Gateway system (Life Technologies) was used to generate the expression vector pUASt-Venus-dRYBP. Transgenic flies were obtained following standard procedures using *white^1118^* flies as host.

### Quantitative real-time PCR (qRT-PCR)

RNA was isolated from 10–15 adult female flies of appropriate genotype. RNA extraction and RT reactions were performed as previously described [31]. qRT-PCR was performed using FastStart Universal SYBR Green MasterRox (Roche) in an Applied Biosystems 7900 Sequence Detector System. Quantified mRNA levels were expressed as relative fold change normalized to *RpL32*. The sequences of the primers and their efficiencies (in brackets) are the following:

*RpL32* Forward: 5′-GACGCTTCAAGGGACAGTATCTG-3′, Reverse: 5′-AAACGCGGTTCTGCATGAG-3′ (1,89); *dRYBP* Forward: 5′-CATGTTGACACCTGGCTCCTG-3′, Reverse: 5′-CGAAGGTGATCGAGGAGAAC-3′ (1,97) ; *Dpt*: Forward: 5′-GCTGCGCAATCGTTCTACT-3′, Reverse: 5′-TGGTGGAGTGGGCTTCATG-3′ (1,98) ; *AttB* Forward: 5′-CCTACAACAATGCTGGTCATGGT-3′, Reverse: 5′-CCTACAACAATGCTGGTCATGGT-3′ (2,03); *Relish* Forward: 5′-TTAGCGTGGCCAACACAATG-3′, Reverse: 5′-GAACTGCCATGTGGAGTGCAT-3′ (1,98); *PGRP-LC* Forward: 5′-GCATTCAATGGTGGTCCCA-3′ Reverse: 5′-CCGGATCTTCGTGTTTGGAG-3′ (1,97); *imd* Forward: 5′-TTCGGCTCCGTCTACAACTT-3′, Reverse: 5′-GTGATCGATTATGGCCTGGT-3′ (2,03); *Dredd* F: 5′-CAAAAGGTGGGCCTCTGCT Reverse: 5′-GTAGGTGGCATCCGAGTGGT-3′ (2,02); *Tab-2* Forward: 5′-TGTCATGGAGGAATGCGATC-3′, Reverse: 5′-GCTTCTGACGCTCGATAGTGG-3′ (1,97); *TAK1* Forward: 5′-GATCTGAGTCCCAGCGAAAGC-3′, Reverse: 5′-CATCGCTCTTTGCGTTCGT-3′ (1,96); *ird-5* Forward: 5′-TAGTGATCCATTGGCGAAACC-3′, Reverse: 5′-GCTTGGTGGCAATTTCACG-3′ (1,96); *skpA* Forward: 5′-CTCCCGAGGAAATACGCAAG-3′, Reverse: 5′-CGGGCGAAAAGTCCTTCTTA-3′ (1,99); *dIAP2* Forward: 5′-ATGCAAGGTATGCTTGGACGA-3′, Reverse: 5′-TGATTGCAGGTGGCCAAGT-3′ (1,90)

### Statistical analysis

The data from all the qRT-PCR experiments represent the mean + SEM of three biological repeats with 10–15 individuals per sample. For inter-assay comparability, values within each experiment were routinely normalized to the wild type or relevant other control at a given time point. Data were analyzed using ANOVA with Bonferroni post-test.

### Immuno-staining

Fat bodies from adult females were dissected in PBS, fixed in 4% paraformaldehyde and stained with either anti-dRYBP antibody (1∶100) [27] and biotinylated anti-rabbit antibody (1∶200) as previously described [31], or with rabbit-anti-GFP (Interchim) followed by Alexa488-anti-rabbit (Molecular Probes) and DAPI. Images were taken on either a Zeiss CDD microscope or a Zeiss confocal microscope with a 40-fold oil-immersion objective.

## Results

### Loss of *dRYBP* results in over-activation of the Imd pathway

**Figure 1:**
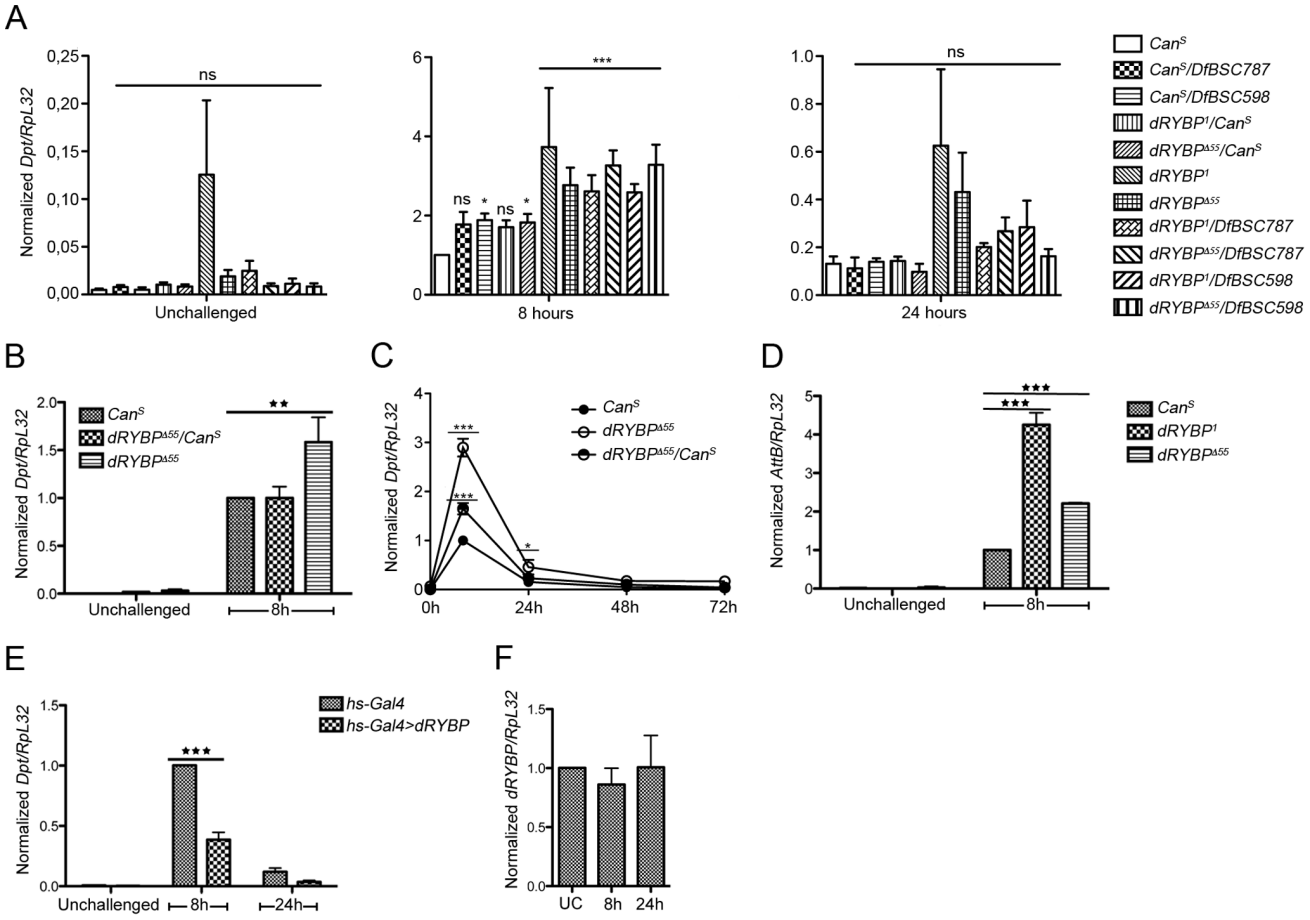
Genetic modulation of *dRYBP* expression levels affects AMP production. (A, B, C, D, E). (**A**) All tested *dRYBP* mutants show an excessive immune response to Gram-negative infection. Adult females of indicated genotypes were left unchallenged (left panel) or infected by pricking with *Ecc15* (heat-killed at 95°C during 15 min) and collected at 8 h (middle panel) and 24 h (right panel) after infection for *Diptericin* (*Dpt*) mRNA quantification. The complete data set (unchallenged, 8 h, 24 h) was analysed by two-way ANOVA with Bonferroni post-tests, using *Can^S^* as control. (**B**) *dRYBP* mutants also show excessive Imd activation after infection with another Gram-negative pathogen, *E. coli*. Adult females of indicated genotypes were treated as in (A). (**C**) Infection time-course in wild-type, heterozygous and homozygous *dRYBP^Δ55^* mutants. Flies were treated as in (A). (**D**) Loss of *dRYBP* affects at least two Imd-dependent antimicrobial peptides. mRNA levels of *Attacin-B* (*AttB*) were quantified after infection as described in (A). (**E**) Overexpression of *dRYBP* reduces the Imd response to Gram-negative infection. Adult females were infected as in (A), then were heat-shocked for 1 h at 37°C and collected at 8 h and 24 h after infection for quantification of *Dpt* mRNA levels. (**F)** *dRYBP* expression levels are not affected by infection. Wild-type adult females were infected as in (A) and *dRYBP* mRNA levels were monitored over time. For all graphs, data represent mean + SEM of at least 3 biological repeats, and asterisks denote the following p values: *, 0.01<p<0.05; **, 0.001<p<0.01; *** p<0.001; ns, not significant.

The phenotypes associated with null alleles of *dRYBP* indicate that this gene plays a role in diverse biological processes [30]. We performed a genetic screen to search for *dRYBP* interacting genes and found a number of components of the Imd pathway (Fig. S1). Therefore, we chose to analyze the possible involvement of *dRYBP* in modulating the immune response to Gram-negative infection. We investigated whether the *dRYBP* gene could be regulating the expression of antimicrobial peptides (AMPs), which are always induced upon activation of the immune response. mRNA expression levels of *Diptericin* (*Dpt*), an AMP normally induced upon activation of the Imd pathway [41], were measured by qRT-PCR in *dRYBP* null adult flies [30]. In uninfected adult *dRYBP* mutant flies, *Dpt* expression was not significantly altered compared to the wild-type control (Fig. 1A, unchallenged). However, upon infection with the Gram-negative bacterium *Erwinia carotovora carotovora 15* (*Ecc15*), *Dpt* expression was significantly increased beyond wild type levels after 8 h of infection in *dRYBP^1^*and *dRYBP^Δ55^* homozygous mutant flies, as well as in flies of *dRYBP^1^* or *dRYBP^Δ55^* genotype over *dRYBP* genomic deficiencies excluding background effects (Fig. 1A, 8 hours). *dRYBP* mutants showed a normal return to baseline at 24h after challenge (Fig 1A, 24 hours). Infection with another Gram-negative pathogen, *E.coli*, gave similar results to *Ecc15* (Fig. 1B). Moreover, the increase in *Dpt* expression was dose dependent since *dRYBP* heterozygous mutant flies showed an intermediate phenotype, suggesting that one dose of dRYBP is insufficient to ensure wild type regulation (Fig. 1A and C). Furthermore, expression of *AttB*, another IMD-dependent AMP [42], was similarly affected by loss of *dRYBP* (Fig. 1D). Taken together, these results indicate that *dRYBP* functions as a negative modulator of the IMD-mediated immune response.

The up-regulation of the *Dpt* expression observed in *dRYBP* mutants prompted us to study whether high levels of dRYBP expression were capable of repressing *Dpt* production. mRNA levels of *Dpt* were measured in *hs-Gal4;UAS dRYBP* flies in the presence or absence of *Ecc15* infection. Fig. 1E shows that dRYBP overexpression significantly reduced *Dpt* expression following 8 h of infection. The reduction in *Dpt* expression in the presence of high levels of dRYBP further supports the notion that *dRYBP* is contributing to the negative regulation of the IMD-pathway mediated immune response.

Several negative regulators of the Imd pathway, including PGRP-LF, Pirk and PGRP-LB, are induced upon Gram-negative infection and form a negative feedback loop [43]–[47]. We therefore tested whether *dRYBP* expression levels in adult flies were affected by infection with *Ecc15*. mRNA levels of *dRYBP*, as measured by qRT-PCR, were not significantly changed after either 8 h or 24 h of infection (Fig. 1F). This result is in agreement with microarray studies [48], [49] and suggests that *dRYBP*-mediated regulation of *Dpt* expression is not controlled at the level of its own transcription.

### Expression of Imd pathway components in *dRYBP* mutant flies

**Figure 2:**
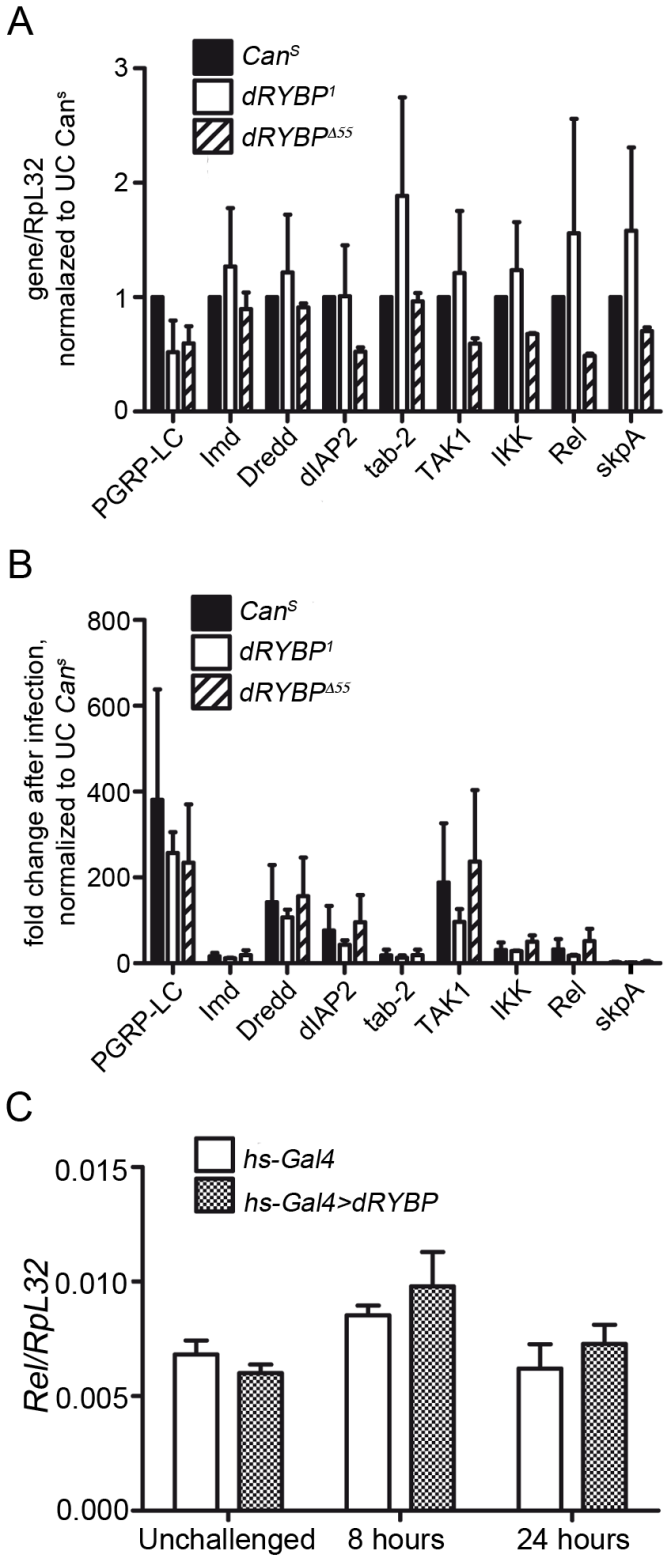
Loss of *dRYBP* does not affect expression of canonical Imd pathway components. (**A)** mRNA levels of indicated Imd pathway components were quantified in wild-type and *dRYBP* mutant flies under unchallenged conditions (A) or (**B**), 8 h after infection by pricking with *Ecc15.* Data in (A) and (B) represent mean + SEM of two pooled biological repeats. For each gene, expression is normalized to expression in unchallenged (uc) *Can^S^*. No significant difference in gene expression between wild-type and *dRYBP* mutants, by two-way ANOVA with Bonferroni post-test. (**C**) Overexpression of *dRYBP* does not affect *Relish* expression. Adult females were infected as in (B), then were heat-shocked for 1 h at 37°C and collected at 8 h and 24 h after infection for quantification of mRNA levels of the IMD-dependent transcription factor *Relish*. Data represent mean + SEM of 3 biological repeats.

Because dRYBP has been suggested to function as a transcriptional regulator together with the Polycomb and trithorax proteins [27], [30] we analyzed whether dRYBP could be involved in the transcriptional regulation of canonical Imd pathway components. For this, we quantified the mRNA levels of selected Imd pathway components in homozygous *dRYBP^1^* and *dRYBP^Δ55^* mutant flies both in unchallenged conditions and after 8 h of infection with *Ecc15*. Expression levels of all Imd pathway components studied were unaffected in *dRYBP* mutant flies compared to control *Can^S^* flies, whether in unchallenged conditions (Fig. 2A) or after 8 h of infection (Fig. 2B). Moreover, we also studied whether over-expression of dRYBP affects the expression of the most downstream component, *Relish*. Fig. 2C shows that high levels of dRYBP do not influence *Relish* expression. These results indicate that *dRYBP*-mediated repression of *Dpt* expression is not due to dRYBP acting directly as a transcriptional repressor on canonical Imd pathway genes.

### dRYBP is expressed in the nucleus of fat body cells

**Figure 3:**
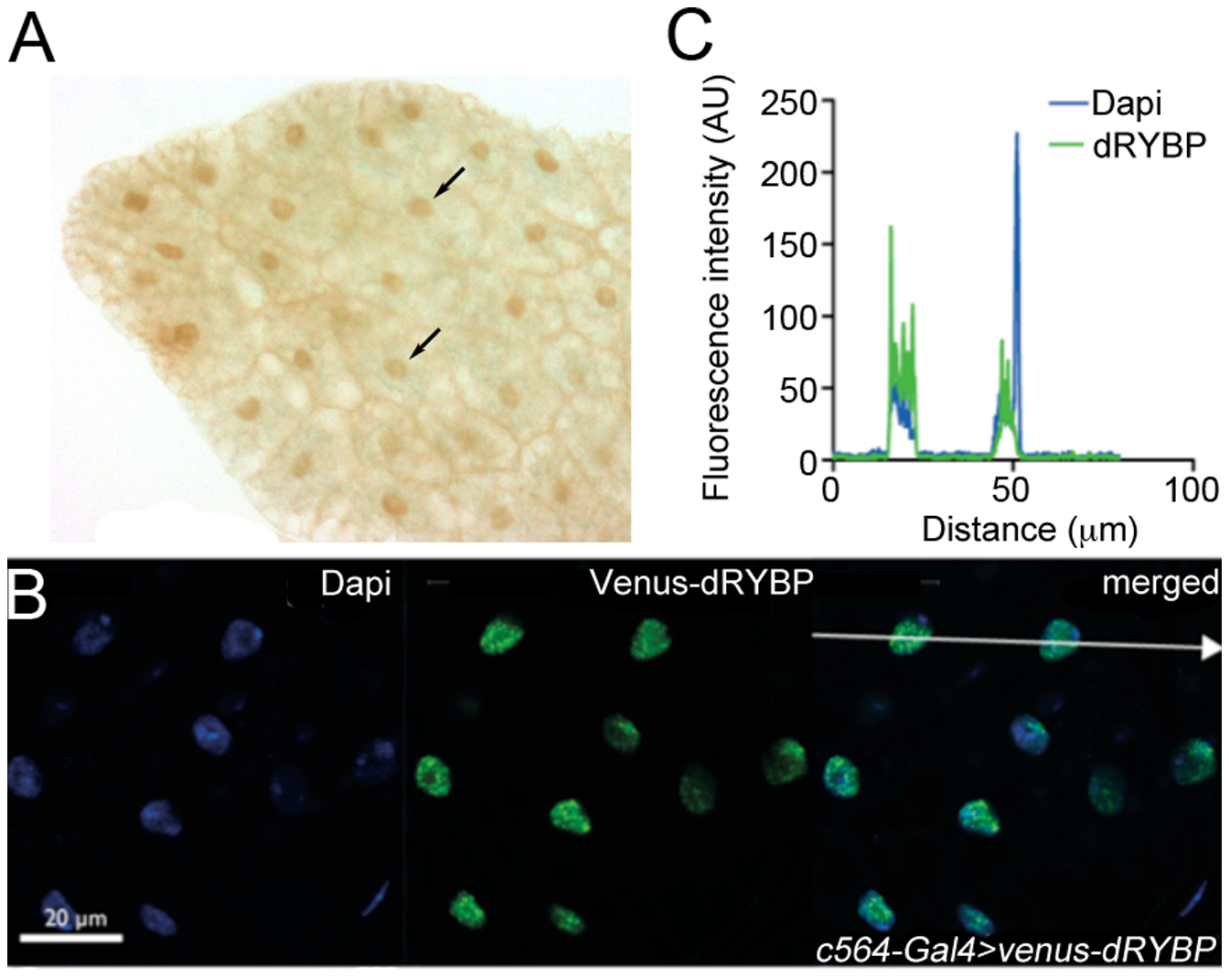
dRYBP localizes to the nuclei of adult fat body cells. (**A**) dRYBP protein is expressed in the nuclei of fat body cells (arrows). (**B**) Flies overexpressing Venus-dRYBP under the fat body specific driver *c564-Gal4* were infected by pricking with *Ecc15*. Fat bodies were dissected 3 h after infection, fixed and stained with anti-GFP antibody and DAPI. Images are representative of several *UAS-Venus-dRYBP* insertion lines. (**C**) Fluorescence profile along arrow in (B) shows dRYBP is exclusively nuclear and excluded from nucleoli. AU (Arbitrary Units). Scale bar denotes 20 µm.

We have previously shown that dRYBP is ubiquitously expressed in the embryo and the imaginal discs of the larva and localizes to the nuclei of cells [27]. We performed immuno-staining with anti-dRYBP antibody (Fig. 3A) in fat body cells and found that dRYBP is also present in the nuclei of this immuno-competent tissue in wild type conditions, which is in agreement with FlyAtlas data [50] (CG12190, http://www.flyatlas.org). Since the Imd pathway includes both cytosolic and nuclear steps, we asked whether dRYBP localized to any cellular compartment in particular during infection. To this aim we constructed *UAS-Venus-dRYBP* flies and used the *c564-Gal4* line to drive Venus-dRYBP expression specifically in the fat body where AMPs are highly up-regulated in response to systemic infection [2]. As shown in Fig. 3B and C, the subcellular localization of dRYBP in adult female fat body cells was exclusively nuclear at 3 h after infection with *Ecc15*, as shown by fluorescence intensity profiles across cells. This suggests that either the putative dRYBP expression in the cytoplasm is too low to be detected using this approach or that dRYBP acts on nuclear Imd pathway components rather than upstream cytosolic adaptors.

### Epistatic relationships between dRYBP and components of the Imd pathway

To delineate the hierarchical positioning of dRYBP in the Imd signaling cascade, we studied whether overexpression of dRYBP could repress *Dpt* expression when the pathway was activated by forced expression of IMD or Relish, both of them activators of the Imd cascade [13], [38], or by decreasing the expression of *skpA*, a known repressor of the Imd cascade [26]. The latter two seemed particularly relevant based on their proven nuclear localization and hence accessibility to dRYBP.

**Figure 4:**
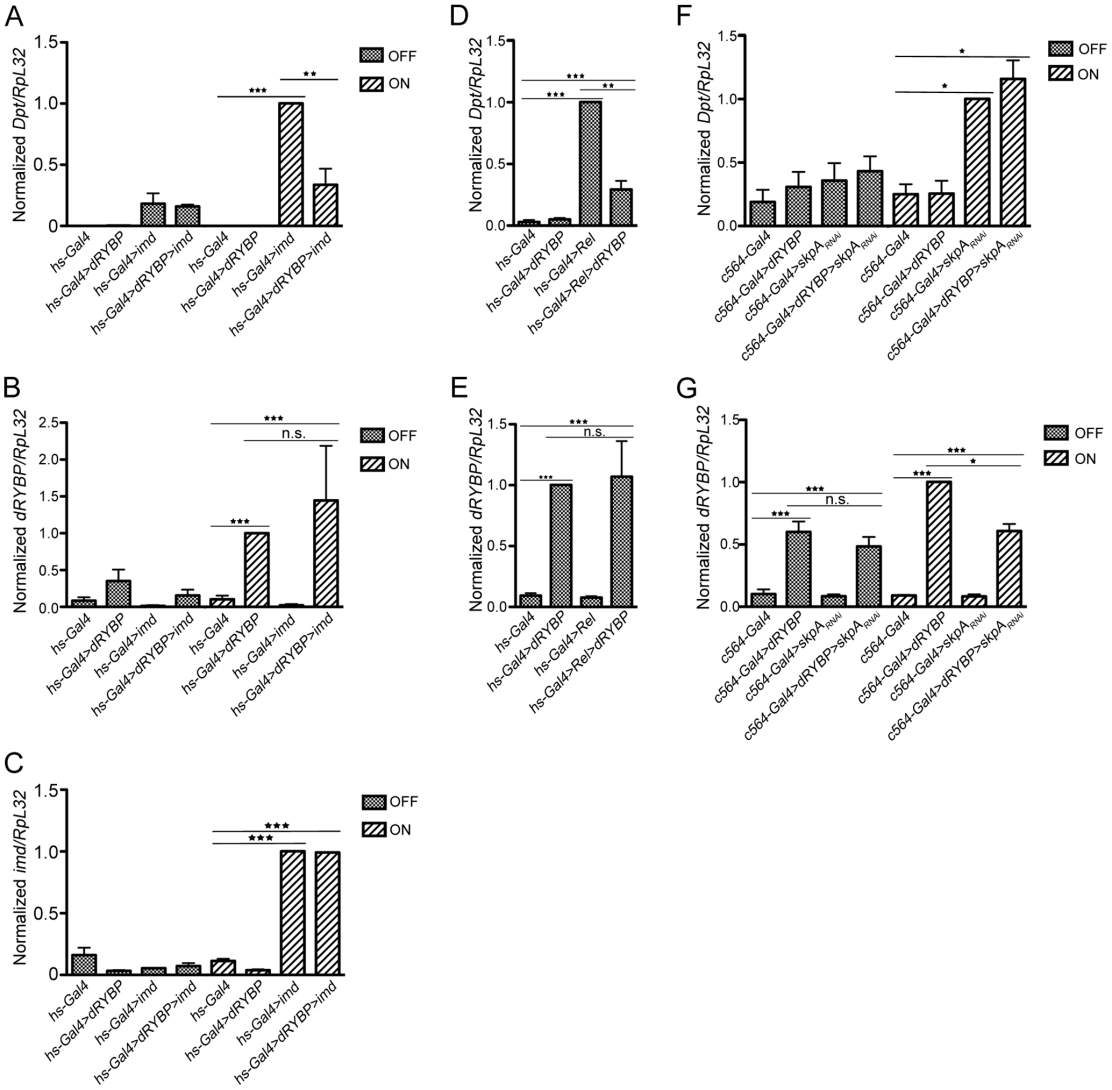
Epistatic relationships between dRYBP and components of the Imd pathway. (**A**) dRYBP acts downstream of IMD. Flies overexpressing *dRYBP*, *imd*, or both under the control of a heat-shock-inducible promoter were kept at 18°C (OFF) or exposed to 37°C for 1 h, then shifted to 29°C for 7 h (ON), at which point *Dpt* mRNA levels were assessed to measure Imd pathway activity. (**B, C**) Overexpression of multiple UAS constructs does not lead to Gal4 dilution. (B) shows qRT-PCR quantification of mRNA levels of *dRYBP* expression. *dRYBP* expression is increased under induced conditions (compare *hs-Gal4 to hs-Gal4;UAS-dRYBP*) and does not significantly change when two UAS constructs are concomitantly expressed (compare *hs-Gal4;UAS-dRYBP* to *hs-Gal4,UAS-imd/UAS-dRYBP*). (C) shows qRT-PCR quantification of mRNA levels of *imd* expression. *imd* expression is increased under induced conditions (compare *hs-Gal4* to *hs-Gal4,UAS-imd*) and does not significantly change when two UAS constructs are concomitantly expressed (compare *hs-Gal4,UAS-imd* to *hs-Gal4,UAS-imd/UAS-dRYBP*). (**D**) dRYBP acts downstream of Relish. Flies overexpressing *dRYBP*, *Rel* (*Relish-His-6*), or both under the control of a heat-shock-inducible promoter were kept at 18°C, which induces low overexpression of all constructs. *Dpt* mRNA levels were assessed as a measure of Relish-dependent transcriptional activation. (**E**) qRT-PCR quantification of mRNA levels of *dRYBP* expression in the same crosses. *dRYBP* expression is increased when overexpressed with *hs-Gal4* in flies kept at 18°C (compare *hs-Gal4* to *hs-Gal4;UAS-dRYBP*) and does not significantly change when two UAS constructs are concomitantly expressed (compare *hs-Gal4;UAS-dRYBP* to *hs-Gal4/UAS-Relish-His-6;UAS-dRYBP*). **(F)** dRYBP acts upstream of SKPA. Flies overexpressing *dRYBP*, RNAi against *skpA*, or both under the control of the fat-body specific driver *c564-Gal4* were either kept at 18°C (OFF) or shifted to 29°C for 24 h (ON), at which point *Dpt* mRNA levels were assessed to measure Imd pathway activity. (**G**) qRT-PCR quantification of mRNA levels of *dRYBP* expression. *dRYBP* expression is increased under induced conditions (compare *c564-Gal4* to *c564-Gal4;UAS-dRYBP*) and does not significantly change when two UAS constructs are concomitantly expressed (compare *c564-Gal4;UAS-dRYBP* to *c564-Gal4;UAS-dRYBP/UAS-skpA_RNAi_*). Asterisks denote the following p values: *, 0.01<p<0.05; **, 0.001<p<0.01; *** p<0.001; ns, not significant. Data represent mean + SEM of 3 biological repeats.

Activation of the Imd cascade by overexpression of IMD in *hs-Gal4,UAS-imd* flies resulted in increased production of *Dpt* (Fig. 4A, compare *Dpt* expression in *hs-Gal4* vs *hs-Gal4,UAS-imd* flies) [38]. Furthermore, in *hs-Gal4,UAS-imd/UAS-dRYBP* flies, *Dpt* expression was repressed (Fig. 4A, compare *Dpt* expression in *hs-Gal4,UAS-imd* vs *hs-Gal4,UAS-imd/UAS-dRYBP*). This inhibition is not due to dilution of the GAL4 protein as the expression levels of both *dRYBP* and *imd* were very similar in flies overexpressing a single gene versus flies overexpressing both genes concomitantly (Fig. 4B, C for controls of the dilution of the GAL4 protein). Therefore, these results indicate that dRYBP functions either together with the IMD protein or downstream of the IMD protein in the regulation of the Imd pathway.

Activation of the Imd cascade by overexpression of Relish in *hs-Gal4/UAS-Relish* flies increased the expression of *Dpt* (Fig. 4D, compare *Dpt* expression in *hs-Gal4* flies to *hs-Gal4/UAS-Relish*) [39]. However, *Dpt* expression was still diminished in *hs-Gal4/UAS-Relish;UAS-dRYBP* flies (Fig. 4D, compare *Dpt* expression in *hs-Gal4/UAS-Relish* to *hs-Gal4/UAS-Relish;UAS-dRYBP* and Fig. 4E for Gal4 dilution). These results indicate that dRYBP functions either together with Relish or downstream of Relish in the repression of the Imdpathway.

Finally, inactivation of *skpA* increased *Dpt* expression (Fig. 4F, compare *Dpt* expression in *c564-Gal4* flies to *c564-Gal4;UAS-skpA_RNAi_*) [26]. However, overexpression of *dRYBP* in these conditions did not affect the expression of *Dpt* (Fig. 4F, compare *Dpt* expression in *c564-Gal4;UAS-skpA_RNAi_* to *c564-Gal4;UAS-skpA*_RNAi_ /*UAS-dRYBP* and Fig. 4G for controls of the dilution of the GAL4 protein). These results indicate that dRYBP functions either together with SKPA or upstream of SKPA to repress the activation of the Imdpathway.

Taken together, our investigation into the epistatic relationships between *dRYBP* and *imd*, *Relish* and *skpA* indicates that dRYBP functions at the level of the ubiquitin E3-ligase SKPA to repress the activation of the Imd pathway and suggest that the ubiquitin-binding dRYBP may function together with SKPA in the degradation of Relish to inhibit the immune response.

## Discussion

A balanced response to infection requires the control of both positive and negative regulation of the Toll and Imd immune signaling pathways [2], [3], [6]. Activation of the pathways to combat the infection through the production of AMPs is as important as their repression since flies rapidly die when several negative regulators of the Imd pathway are simultaneously deleted [51]. In the last decades, investigations into the mechanisms of immune responses in *Drosophila* have mainly focused on deciphering the activation of AMP production. However, more recently research interest has switched to inhibitors of the pathway, and with the identification of multiple candidates a picture of the mechanisms controlling negative regulation is emerging [6], [20], [23]–[25], [43]–[46], [52]. Here we have introduced the *dRYBP* gene as a novel player in the negative regulation of the *Drosophila* Imdpathway.

The dRYBP protein and its vertebrate ortholog RYBP/DEDAF/YAF2 have been shown to interact with a diverse range of proteins [27]–[30], [33], [34], [53]. Both the fly and the vertebrate proteins have been found to interact genetically and molecularly [27]–[30], [34] with the Polycomb group of proteins that maintain the repressed gene transcriptional states by epigenetic mechanisms (for recent reviews [54], [55]). Our investigation into the mechanisms by which dRYBP is acting on the Imd pathway indicates that dRYBP does not control the expression of the canonical Imd pathway genes transcriptionally (Fig. 2) suggesting that dRYBP does not function as a Polycomb group protein in the regulation of the immune response.

Notably, the dRYBP protein contains a ubiquitin binding domain and vertebrate RYBP/DEDAF/YAF2 has been shown to bind ubiquitylated proteins [28]. An increasing number of both activating and regulatory Imd pathway components involve ubiquitin-dependent modifications [16], [19], [20], [26]. It seemed therefore likely that dRYBP might exert its regulatory role through interaction with proteins that are either ubiquitylated themselves or play a role in ubiquitylation. A search for dRYBP-interacting proteins by mass spectrometry (Simón *et al.*, manuscript in preparation and Fig. S2A) has shown that in unchallenged conditions the dRYBP protein physically interacts with CULLIN-1 and SKPA proteins, both members of the E3-ubiquitin ligase SCF complex that targets substrates to the 26S proteasome and plays a pivotal role in regulating diverse developmental events [56]–[59]. Members of the SCF-E3 ubiquitin ligase complex, in particular SKPA, CULLIN-1 and SLIMB, have been previously reported to function as repressors of AMP production in uninfected flies [26] and proposed to repress the Imd pathway by promoting the ubiquitylation and subsequent degradation of a constitutively active Relish protein, as well as down-regulating AMP production after infection in vivo [26]. Our epistatic genetic interaction analysis (Fig. 4) demonstrates that dRYBP functions either together with or downstream of Relish, and together with or upstream of SKPA to dampen the activated immune response. Based on the epistatic and proteomic data, it is likely that dRYBP associates with the SCF-E3 ubiquitin ligase complex to inhibit the activated immune response at the level of Relish degradation.

We propose that dRYBP functions as a ubiquitin adaptor protein in the regulation of the Imd pathway. dRYBP may contribute to the termination of Imd pathway activation by promoting assembly of the SCF complex, which is known to ensure degradation of Relish [26]. Of note, the observed haploinsufficiency of *dRYBP* (Fig. 1) would suggest that complex assembly is sensitive to the stoichiometry of its components. However, the fact that dRYBP null mutants show a normal return to baseline suggests that dRYBP is non-essential in shutting off signaling, and contributes to the amplitude rather than the duration of signaling output. In the complete absence of dRYBP, failure to assemble the SCF complex correctly and timely would lead to transient accumulation of activated nuclear Relish and to excessive transcription of Relish-dependent genes.

The vertebrate homolog of Relish, the NF-κB subunit p105 protein, has been demonstrated both to be ubiquitylated and to belong to the superfamily of DD (Death Domain) containing proteins [60]. The latter plays important roles in the assembly and activation of apoptotic and inflammatory complexes [36]. Curiously, the *Drosophila* RYBP protein and the vertebrate RYBP/DEDAF/YAF2 protein have been shown to interact with Death Effector Domain (DED) proteins, a subfamily of the DD proteins [31], [32], [36]. A search for a DD domain in the Relish protein using the Death Domain Database (www.deathdomain.org) [61] returned that indeed Relish contains a putative DD domain with a relevant percentage of similarity to the DD in mammalian p105 (Fig. S2B, C) [62]. Possibly, the interaction between Relish and dRYBP involves both the DD binding domain and ubiquitin signatures on Relish to promote its proteasomal degradation through the SKPA/Cullin complex. It now remains to be clarified whether and how dRYBP interacts with Relish upon infection and whether dRYBP has an effect on the degradation of Relish. At this stage, we cannot exclude that dRYBP might also function at other steps in this pathway, including in the process of ubiquitylation of the IMD protein [17], [19], to negatively regulate the immune response.

The present work has shown that *dRYBP* contributes to the regulation of the fly immune response, a fundamental systemic organism reaction to overcome external harm or stress. The decision to repress the activated immune response likely depends on the nature of the stress signal. Further investigations will shed light on the contribution of dRYBP in reaching a balanced immune response and will reveal whether the evolutionarily conserved RYBP protein may have a novel function in the control of the human innate immune response.

## Supporting Information

Figure S1 The *dRYBP* loss of function phenotype is modulated by mutant alleles of the Imd pathway. (**A**) Crossing scheme. The *engrailed*-*Gal4* (*en*-*Gal4*) driver was used to express UAS-dRYBP_RNAi_ and UAS-VDRC_RNAi_ in the wing. Female virgins *en-Gal4*/*CyO;UAS-dRYBP_RNAi_ /MKRS* were crossed with males *UAS-VDRC_RNAi_* and maintained at 29°C. From these crosses, the *en-Gal4;UAS-VDRC_RNAi_* progeny were analyzed for wing phenotypes associated with the particular *VDRC_RNAi_* line under study and the *en-Gal4;UAS-dRYBP_RNAi_,/UAS-VDRC_RNAi_* progeny were analyzed for the penetrance of the wing blister phenotype. (**B**) Wild-type wing. (**C**) *en-Gal4>dRYBP_RNAi_* wing showing a blister (arrow) in the posterior compartment. (**D**) Quantification of flies with wing blisters of the indicated genotypes. *en-Gal4>dRYBP_RNAi_/UAS-GFP* was used as a control. Importantly, this screen merely shows genetic interaction between dRYBP and Imd pathway components. It is not yet clear why the penetrance of blister phenotype in our screen can be modulated by mutations in genes involved in the innate immune response or why the penetrance is modulated by mutations in both activators and repressors of the Imd pathway. The wing phenotypes are probably due to these factors involved in other biological processes [38], [63].
(TIF)

Figure S2 Mass spectrometry data and localization of a Death Domain in the Relish protein**.** (**A**) Mass spectrometry results showing the scores for the indicated dRYBP interacting proteins. (**B**) Alignment of Human p105 and *Drosophila* Relish protein sequence. Indicated in green is the predicted Death Domain (DD) sequence (www.deathdomain.org). (**C**) Magnification of the predicted DD domain. (**D**) Alignment of other DD containing proteins with the predicted DD domain in the Relish proteins.
(TIF)
