# Supplementary figures and images for: dRYBP Contributes to the Negative Regulation of the Drosophila Imd Pathway
Source: PLoS One. 2013 Apr 15;8(4):e62052. doi: 10.1371/journal.pone.0062052 (PMC3626645; doi:10.1371/journal.pone.0062052)

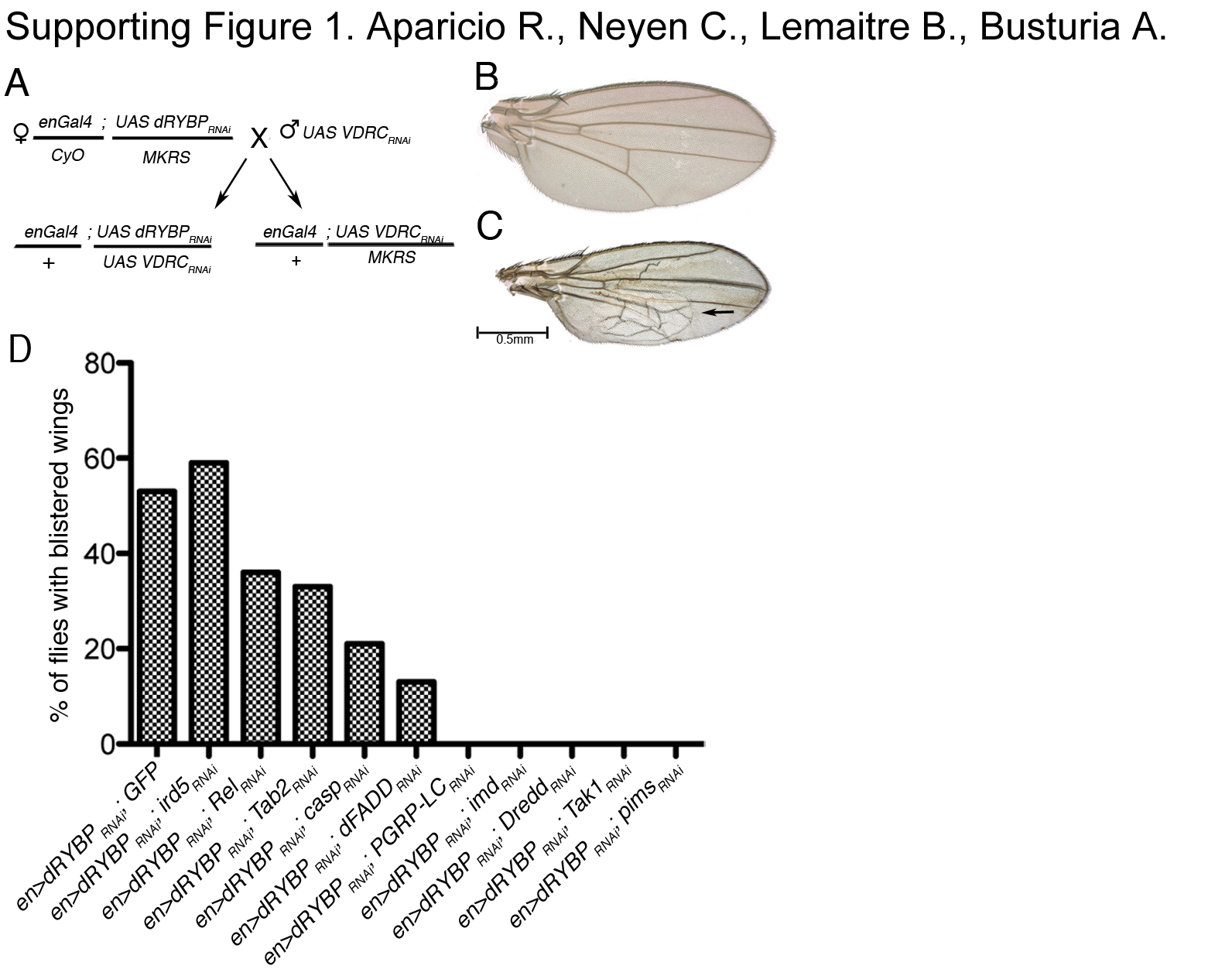

Supplement: Figure S1 — The dRYBP loss of function phenotype is modulated by mutant alleles of the Imd pathway. (A) Crossing scheme. The engrailed-Gal4 (en-Gal4) driver was used to express UAS-dRYBPRNAi and UAS-VDRCRNAi in the wing. Female virgins en-Gal4/CyO;UAS-dRYBPRNAi /MKRS were crossed with males UAS-VDRCRNAi and maintained at 29°C. From these crosses, the en-Gal4;UAS-VDRCRNAi progeny were analyzed for wing phenotypes associated with the particular VDRCRNAi line under study and the en-Gal4;UAS-dRYBPRNAi,/UAS-VDRCRNAi progeny were analyzed for the penetrance of the wing blister phenotype. (B) Wild-type wing. (C) en-Gal4>dRYBPRNAi wing showing a blister (arrow) in the posterior compartment. (D) Quantification of flies with wing blisters of the indicated genotypes. en-Gal4>dRYBPRNAi/UAS-GFP was used as a control. Importantly, this screen merely shows genetic interaction between dRYBP and Imd pathway components. It is not yet clear why the penetrance of blister phenotype in our screen can be modulated by mutations in genes involved in the innate immune response or why the penetrance is modulated by mutations in both activators and repressors of the Imd pathway. The wing phenotypes are probably due to these factors involved in other biological processes [38], [63]. (TIF) [file pone.0062052.s001.tif]

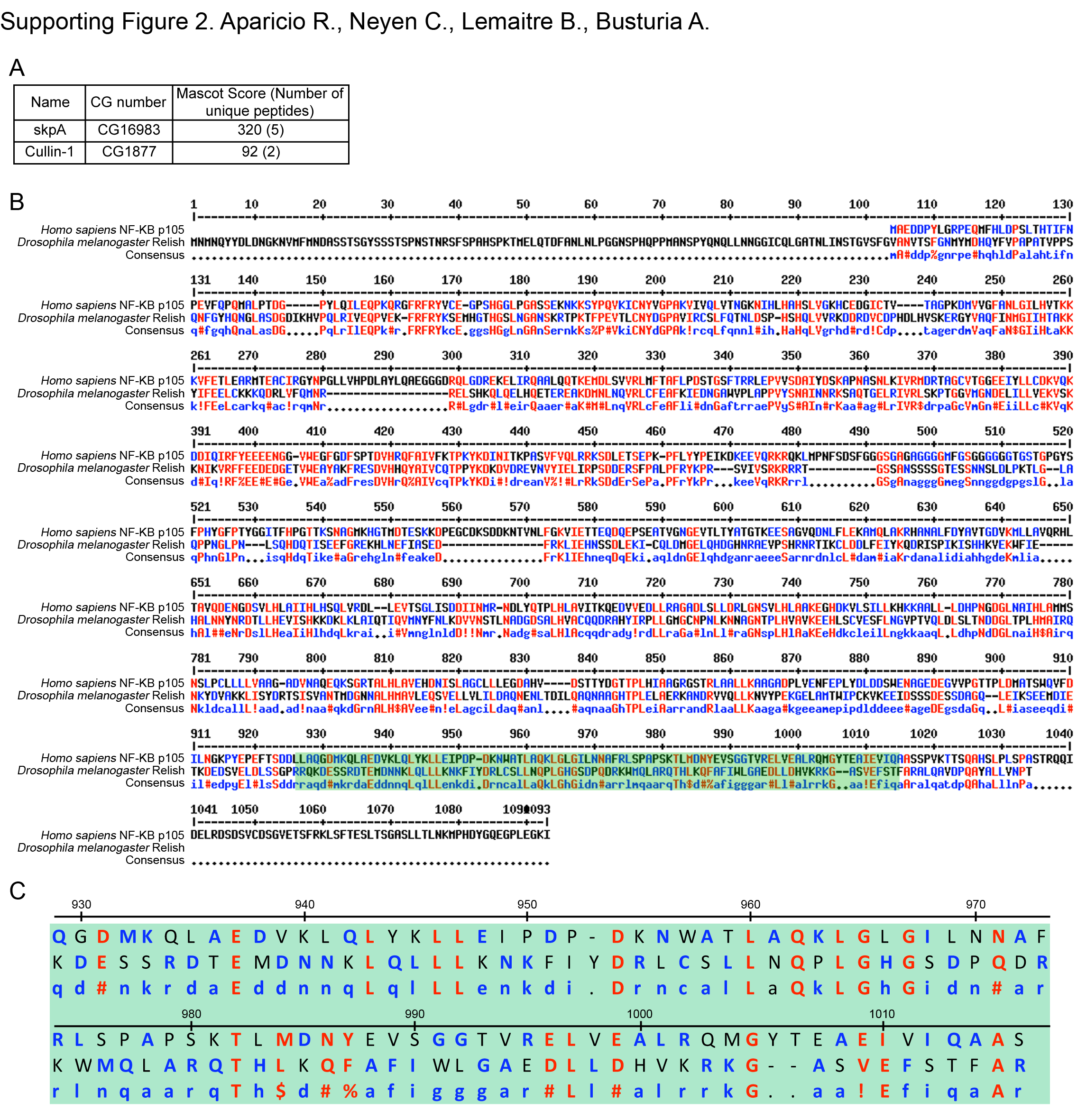

Supplement: Figure S2 — Mass spectrometry data and localization of a Death Domain in the Relish protein. (A) Mass spectrometry results showing the scores for the indicated dRYBP interacting proteins. (B) Alignment of Human p105 and Drosophila Relish protein sequence. Indicated in green is the predicted Death Domain (DD) sequence (www.deathdomain.org). (C) Magnification of the predicted DD domain. (D) Alignment of other DD containing proteins with the predicted DD domain in the Relish proteins. (TIF) [file pone.0062052.s002.tif]
